# Rapid diagnosis of alveolar echinococcosis from lung puncture sample using metagenomic next-generation sequencing: a case report

**DOI:** 10.1186/s12879-024-09553-0

**Published:** 2024-07-09

**Authors:** Chuanlin Zhou, Chunhong Li, Zhenfeng Deng, Xuexin Yan, Li Feng, Zhen Yang, Yanyan Lu, Yinglong Shi, Ke Wang, Jing Luo, Jinliang Kong

**Affiliations:** 1 Ward of Pulmonary and Critical Care Medicine, The First Affiliated Hospital of Guangxi Medical University, Nanning, People’s Republic of China; 2 Infection Diagnosis Center, Guangxi KingMed Diagnostics, Nanning, People’s Republic of China

**Keywords:** Alveolar echinococcosis, Metagenomic next-generation sequencing, *Echinococcus multilocularis*, Diagnose, Case report

## Abstract

**Introduction:**

Alveolar echinococcosis (AE), caused by the larval forms of *Echinococcus multilocularis*, is a zoonotic disease affecting the liver, lungs, lymph nodes, kidneys, brain, bones, thyroid, and other organs. Diagnosing AE in a non-endemic area is usually challenging. With the rapid development and increasing application of sequencing techniques in recent years, metagenomic next-generation sequencing (mNGS) has become a powerful tool for diagnosing rare infectious diseases.

**Case Presentation:**

A 45-year-old woman was admitted to the hospital for the presence of pulmonary shadows for more than 3 months. The lung computed tomography (CT) at a local hospital revealed scattered solid and quasi-circular nodules in the left upper lobe, left lower lobe, right middle lobe, and right lower lobe. The largest nodule was located in the dorsal part of the right lung, measuring 2.0 × 1.7 × 1.5 cm. Moreover, abdominal CT revealed one space-occupying lesion each in the left and right lobes. The pathological analysis of the lung biopsy specimen revealed infiltration of lymphocytes, plasma cells, and eosinophils in the alveolar wall and interstitial area. No pathogenic bacteria were observed in the sputum smear and culture tests. There were no parasite eggs in the stool. The mNGS of the lung puncture tissue revealed 6156 sequence reads matching *E. multilocularis*; thus, the condition was diagnosed as AE. Albendazole 400 mg was administered twice daily, and the patient was stable during follow-up.

**Conclusion:**

This case emphasizes the role of mNGS in diagnosing AE. As a novel, sensitive, and accurate diagnostic method, mNGS could be an attractive approach for facilitating early diagnosis and prompt treatment of infectious diseases, especially when the infection was caused by rare pathogens.

## Introduction

Echinococcosis is a zoonotic disease in which humans serve as one of the intermediate hosts. Humans may be infected by drinking polluted water or eating food contaminated by the feces of definitive hosts [1]. Currently, the four recognized pathogenic species in humans are *Echinococcus granulosus, Echinococcus multilocularis, Echinococcus oligarthrus, and Echinococcus vogeli.* Most infestations in humans are caused by *E. granulosus*, whereas exposure to *E. multilocularis* is less common. *Echinococcus* can cause cystic echinococcosis (CE), alveolar echinococcosis (AE), or mixed echinococcosis. Whereas AE is endemic in the Northern Hemisphere, mainly China, North America and Central Europe, CE is distributed throughout the world [2, 3]. AE results from infection by the larval forms of *E. multilocularis*, which colonizes in the small intestine of a definitive host (dog, fox and other canines). Once an intermediate host (rodents, humans, etc.) ingests the eggs which were released into nature in the faeces of a definitive host, the larvae are liberated in the duodenum and gradually develop into multilocularis cysts [2, 4, 5]. AE can affect various human organs, particularly the liver, lungs, lymph nodes, kidneys, brain, bones, and thyroid [6]. Clinical diagnosis of AE is confirmed by serology, imaging, and pathology [2]. With the rapid development and increasing application of sequencing techniques in recent years, metagenomic next-generation sequencing (mNGS) enables the simultaneous identification of all potential pathogens (bacteria, viruses, fungi and parasites) due to its unbiased sampling [7] and has become a powerful tool for AE diagnosis [8, 9]. We present the case of a patient with multiple solid space-occupying lesions in the lungs and liver, which was diagnosed as AE using mNGS, suggesting the promising application of mNGS in the clinical diagnosis of echinococcosis. The patient was successfully treated with systemic anthelmintic therapy and discharged.

## Case presentation

**Fig. 1:**
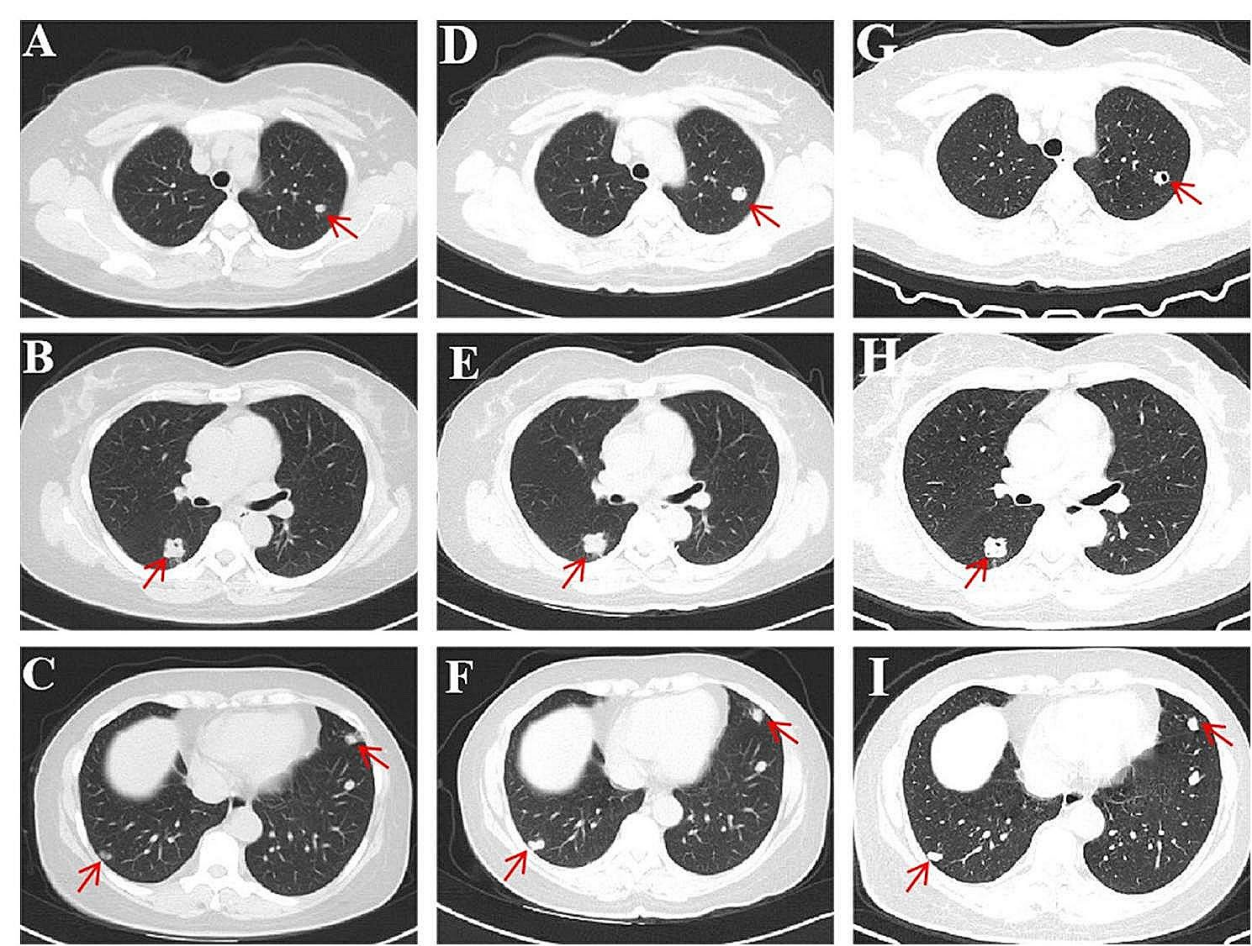
Lung computed tomography images of the patient. A lung CT performed on 3 months prior to admission, shows scattered solid and quasi-circular nodules in the left upper lobe, left lower lobe, right middle lobe, and right lower lobe (**A**-**C**). The largest nodule is seen in the dorsal part of the right lung, measuring 2.0 × 1.7 × 1.5 cm, accompanied by lobulated lesions with slight or moderate heterogeneous enhancement (**B**). A lung CT performed on 5 days prior to admission, shows that the two lung lesions have become larger than before (**D**-**F**). A lung CT performed on 2 months after hospital discharge, indicates that the lesions have shrunk slightly after albendazole therapy (**G**-**I**).

**Fig. 2:**
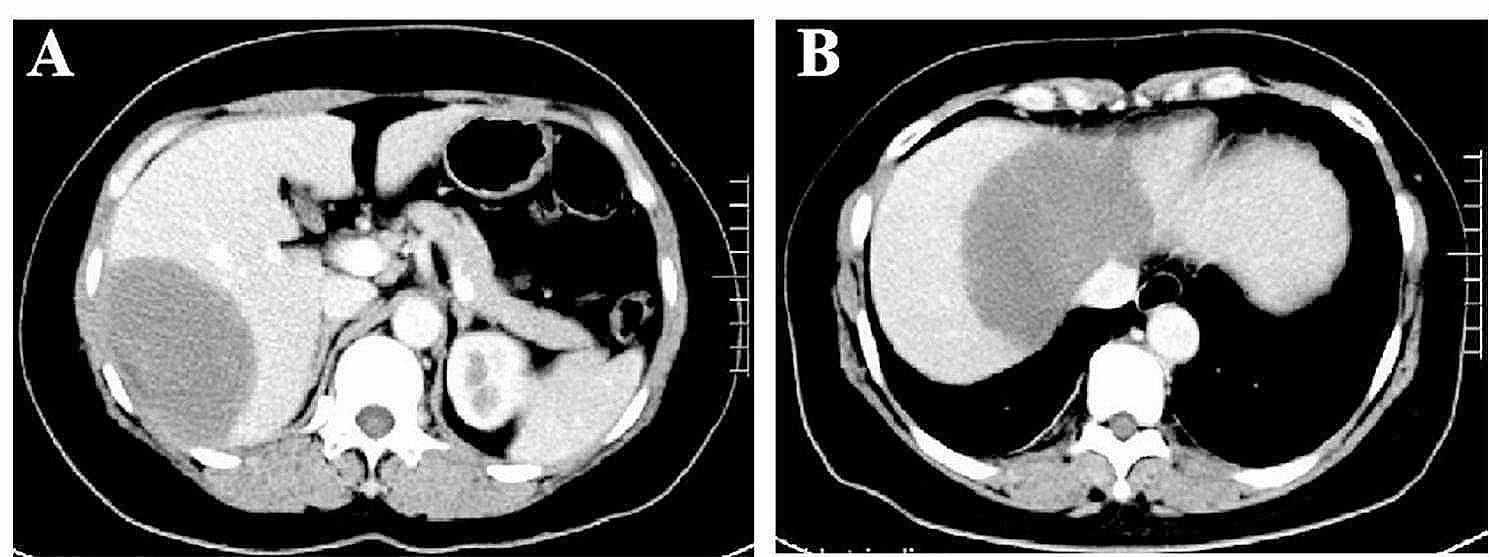
Abdominal enhanced computed tomography of the patient. The abdominal computed tomography performed on 5 days prior to admission, shows one space-occupying lesion each in the right lobe (6.4 × 9.0 × 5.0 cm) (**A**) and left lobe (**B**)

A 45-year-old woman was admitted to the Department of Respiratory Medicine of the First Affiliated Hospital of Guangxi Medical University with the chief complaint of pulmonary shadows present for more than 3 months. Three months before admission, the patient was hospitalized in local hospital due to a lung and liver mass found by examination. The lung computed tomography (CT) at the local hospital revealed scattered solid and quasi-circular nodules in the left upper lobe, left lower lobe, right middle lobe, and right lower lobe. The largest nodule was located in the dorsal part of the right lung, measuring 2.0 × 1.7 × 1.5 cm, accompanied by lobulated lesions with slight or moderate heterogeneous enhancement (Fig. 1A-C). Moreover, bronchoscopic manifestations included endobronchitis, whereas pathological analysis of the biopsy sample obtained through the fiberoptic bronchoscope indicated chronic inflammation with no malignant changes. The puncture biopsy of the lung neoplasms was performed under CT guidance. The histopathology revealed chronic inflammatory changes in the lesions, with a small amyloid deposition and no obvious heterotype. The patient did not receive further treatment because of no obvious symptoms, and she asked to be discharged and was told to re-examine imaging after 3 months. However, 5 days before admission, a re-examination lung CT at the local hospital revealed that the two lung lesions had grown larger than before (Fig. 1D-F). Meanwhile, the abdominal CT revealed one space-occupying lesion each in the left and right lobes, with the larger mass located in the latter (6.4 × 9.0 × 5.0 cm). The contrast-enhanced CT image suggested the presence of a benign liver mass; hence, liver hemangioma could not be excluded (Fig. 2). For further diagnosis, the patient visited our hospital to assess the exact nature of the lung lesions.

(CT, computed tomography)

The patient had engaged in dog slaughtering for more than 10 years without wearing masks, gloves, or other protection gear. Some of the dogs slaughtered were transported from other places. The patient was resident in the local city for a long time and had no history of travel to known endemic areas. The patient did not report any specific diseases or a remarkable family history. Physical examination at the time of admission revealed no abnormalities; the body temperature was 36.3 °C; pulse, 103 beats/min; respiratory rate, 20 breaths/min; and blood pressure, 129/92 mmHg. No abnormal findings were observed on neurological examination.

**Fig. 3:**
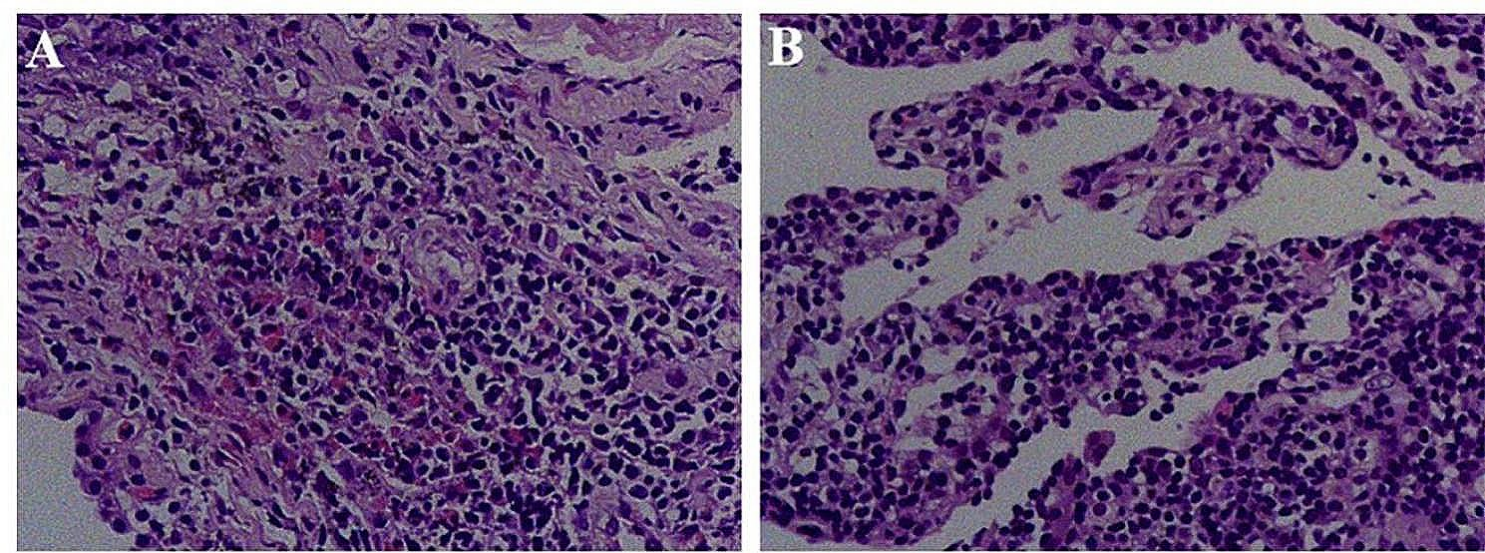
Pathological characteristics of the biopsy sample of the patient. Pathological examination of the lung biopsy sample conducted on 14 days after admission, shows inflammatory lesions and a slightly thickened alveolar septum. The alveolar wall and interstitial area show infiltration of lymphocytes, plasma cells, and eosinophils (**A**, **B**)

Laboratory examinations on admission showed a normal white blood cell count (6.61 × 10^9^/L), normal neutrophil percentage (68.7%), decreased lymphocytes percentage (18.3%; normal range: 30–50%), increased eosinophil absolute value (0.550 × 10^9^/L; normal range: 0.02–0.52 × 10^9^/L), increased erythrocyte sedimentation rate (30 mm/h; normal range: 0–20 mm/h), increased high sensitivity C-reactive protein level (5.8 mg/L; normal range: 0–5 mg/L), and normal procalcitonin level (0.027 ng/mL). Liver function test results for alkaline phosphatase, total bilirubin, and alanine aminotransferase were normal. No pathogenic bacteria were observed in the sputum smear and culture tests. There were no parasite eggs in the stool. The serum (1,3)-β-D glucan and galactomannan test, cryptococcal capsular polysaccharide antigen test, *Mycobacterium tuberculosis* DNA test, and *Mycobacterium tuberculosis* antibody test were all negative. The plasma alpha-fetoprotein value (2.97 ng/mL) was normal. The enzyme-linked immunosorbent assay results for the seven autoantibodies (P53, PGP9.5, SOX2, GAGE7, GBU4-5, MAGE, and CACE) of lung cancer showed increased levels of P53 (30.8 U/mL; normal range: 0–13.1 U/mL), PGP9.5 (18.4 U/mL; normal range: 0–11.1 U/mL), SOX2 (18.0 U/mL; normal range: 0–10.3 U/mL), and GBU4-5 (9.6 U/mL; normal range: 0–7.0 U/mL). No remarkable results were found in the tests for squamous cell carcinoma-related antigen, glycosyl antigen CA153, and CA199. Abdominal color Doppler ultrasound revealed two focal lesions in the liver and a right renal cyst. Chest and upper abdomen CT and enhanced CT revealed multiple solid nodules in both lungs and space-occupying lesions in the liver. Analysis of the lung puncture biopsy sample at an external hospital revealed chronic inflammation, mainly showing B-lymphocyte and plasma cell infiltration. Tracheoscopy was performed again, and a few chronic inflammatory cells had infiltrated the bronchial epithelium, with no obvious exudation in the alveolar spaces. Pathologic analysis of the mucosal biopsy indicated chronic inflammation of the bronchial epithelium, with no evidence of tuberculosis or tumor. Notably, pathologic analysis revealed CD20-positive B cells in the tracheal mucosa; hence, a bone marrow biopsy was performed to rule out lymphoma and other blood diseases. On day 14 after admission, a lung biopsy was performed. Pathological examination indicated inflammatory lesions, and the alveolar septum was slightly thickened. Moreover, infiltration of lymphocytes, plasma cells, and eosinophils was observed in the alveolar wall and interstitial area (Fig. 3).

**Fig. 4:**
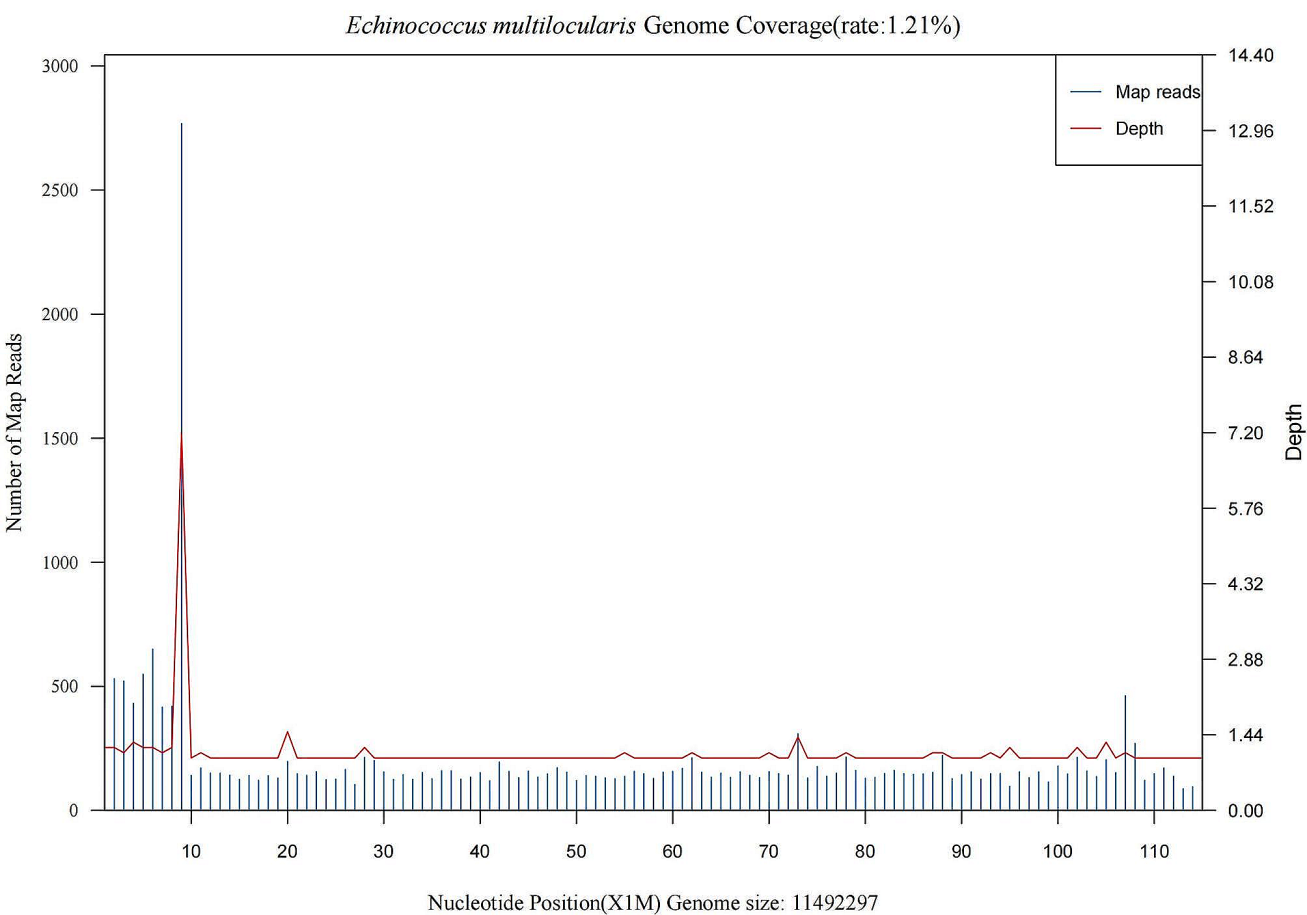
Metagenomic next-generation sequencing results of the patient’s lung puncture sample. Coverage graph of *E. multilocularis*: The abscissa displays the genome position, the left ordinate displays the number of reads of the pathogen aligned on the genome location (number of map reads shown in blue), and the right ordinate displays the average sequencing depth at the corresponding position (depth, red line in the figure)

On day 15 after admission, the lung puncture tissue was subjected to mNGS for an accurate and rapid diagnosis. The mNGS was conducted on the Illumina Nextseq platform and bioinformatics analysis methods were in accordance with the reported literature [10, 11]. After 22 h, the mNGS results confirmed the presence of *E. multilocularis* in the lung. There were 6156 reads detected for *E. multilocularis* in the lung puncture tissue with genome coverage of 1.21% and species relative abundance of 90.09% (Fig. 4). The mNGS results were further confirmed by the positive result for serum hydatid immunoglobulin G antibody. To further identify with malignant tumor, exclude the coexistence of tumor and infection and determine whether lesions occur in other parts of the body, positron emission tomography/CT examination was perfomed after mNGS with the consent of the patient and her familes. It was revealed multiple nodules in both lungs, which were considered inflammatory nodules, and a possibility of benign space-occupying lesions in the liver. The patient refused to undergo a liver biopsy and it was arduous to clarify the liver pathological characteristics further. Considering the CT characteristics of the patient’s liver and lungs and pathologic results of the lung biopsy sample, the condition was finally diagnosed as AE. The patient was discharged 21 days after admission and was treated conservatively with oral albendazole 400 mg twice a day.

After albendazole treatment for 2 months, the re-examination lung CT revealed that the lesions had shrunk slightly, with partial cavity changes (Fig. 1G-I). Radiofrequency ablation was performed for the treatment of liver lesions 1 months and 2 months after hospital discharge, respectively. The lung lesions continued to decrease in size, and the liver lesions had diminished. Moreover, the routine complete blood count and liver function tests showed no abnormalities. Notably, the patient was instructed to follow up regularly to observe the condition changes and effect of drug administration.

## Discussion and conclusions

AE is a zoonotic disease caused by the tapeworm eggs of *E. multilocularis*. The incidence of AE is lower than that of CE caused by *E. granulosus*. *E. multilocularis* is a parasite mostly found in foxes, followed by dogs, wolves, and cats, and the intermediate hosts are mice. Human beings can be infected by ingesting water or livestock contaminated by the eggs of *E. multilocularis*. AE mainly occurs in high-altitude cold regions dominated by animal husbandry and is endemic in the Mediterranean region, South America, Australia, and northwestern China [12]. The feces of animals infected with *E. multilocularis* contain the echinococcus eggs, and the cold weather helps the survival of the parasite eggs. In some pastoral areas, animals are still fed raw animal viscera, which in turn contributes to the transmission of *E. multilocularis* between animals [13]. Our patient lived in a non-echinococcosis endemic area, and she had never traveled to any endemic area. Notably, she had been engaged in dog slaughtering for a long time without wearing protective equipment, and some of the slaughtered dogs belonged to other regions. The disease is extremely rare in the Guangxi Zhuang autonomous region, and this patient was considered a sporadic case. Currently, no relevant regional epidemiological investigation has been conducted. Considering the patient’s slaughterhouse occupation, it was inferred that she was infected due to contact with the dogs from the endemic areas. The larvae of *E. multilocularis* can invade the portal vein system through the intestinal mucosa, transfer to the liver, and then reach the human circulatory system. Therefore, hepatic involvement is the most common clinical manifestation of AE. With the development of the disease, it can metastasize to the lungs, brain, bones, kidneys, thyroid, and other organs. AE can form massive or nodular solid space-occupying lesions in the liver, which are occasionally seen in the lungs, kidneys, brain, and other organs. The lesions comprise a large number of small and hard vesicles with bean curd-like worm fragments and cystic fluid, without a complete envelope. The margin between the lesions and surrounding tissues remains unclear. Moreover, it can invade the blood vessels and lymphatic vessels, similar to cancer spreading, and lead to distal dissemination and metastasis, making it difficult to distinguish it from malignant tumors. *E. multilocularis* can induce organ failure and obstruction of the tract tissue by direct invasion, mechanical compression, or toxic reaction. Liver involvement can commonly result in liver failure, jaundice, portal hypertension, and esophageal and gastric varices, which could seriously threaten the patient’s life; thus, the lesions and consequences of AE are more severe than those of CE. This patient’s CT showed multiple masses and nodules in the lungs and liver. Furthermore, enhanced CT at varying degrees of enhancement showed that the lesion was lobulated; hence, it is often misdiagnosed as a malignant tumor or metastatic tumor [14]. Although the patient refused to undergo a liver biopsy and it was arduous to clarify the liver pathological characteristics further, considering the CT characteristics of the patient’s liver and lungs and pathologic results of the lung biopsy sample, lung metastasis was conclusively excluded, and the condition was finally diagnosed as AE.

Previously, AE has been diagnosed mainly based on epidemiological history, imaging, immunology, and pathology [9]. However, with the frequent circulation of people and animals, radiologists have limited experience in diagnosing rare endemic diseases, which makes it relatively difficult to diagnose the disease in non-endemic areas, and it may be misinterpreted as a metastatic tumor. Immunological cross-reactions occur in immunology, and it is impossible to determine whether it is a past or recent infection and the species-level identity of the tapeworm. Pathological diagnosis requires a large bulk of pathological tissue and experienced pathologists. Moreover, histopathological biopsy may fail to detect hydatid tissue for diagnosing the disease, which mainly relies on serology and imaging systems [15]. Therefore, conventional diagnostic methods make it extremely challenging to detect and diagnose AE in non-endemic areas. Without prior presumption, mNGS enables rapid detection and comprehensive identification of pathogens, including rare ones. When the pathological diagnosis of this patient remained unclear, mNGS of the punctured tissue indicated the specific pathogen type and gene fragment sequence of *E. multilocularis*, and the condition was finally diagnosed as AE. Echinococcosis usually manifests as multiple organ involvement with imaging features similar to those of metastatic malignant tumors, consequently leading to misdiagnosis. Two cases of AE were previously diagnosed using mNGS to determine the pathogenic gene fragment [5, 6]. A definitive diagnosis of AE was made in a case involving the liver, lungs, and brain, which was determined by the mNGS of the cerebrospinal fluid [9]. It is suggested that the next-generation sequencing of relevant body fluid specimens can also be applied to diagnose echinococcosis. The initial phase of primary AE infection is asymptomatic and may persist for 5–15 years [16], which makes it extremely difficult to diagnose. Similarly, there were no obvious pulmonary symptoms in this case. When conventional tests fail to elucidate the etiology of the infection, mNGS may serve as a new powerful tool to diagnose rare infectious diseases. It also has unique advantages in identifying parasitic diseases. However, the accuracy of mNGS requires further validation in large-sample studies.

The early treatment of AE is totally removing the parasitic lesion through radical surgery combined with anthelmintic therapy [17]. Although surgical treatment is often performed in patients with limited lesions, relevant reports have suggested liver transplantation as the therapy for patients with extensive lesions diagnosed as liver echinococcosis and in those with late-stage AE [5, 18]. Also, Relevant reports indicating that thermal ablation techniques such as radiofrequency or microwave ablation can be applied to treat hepatic AE [19, 20], but debates are still ongoing with respect to its treatment. Without surgical removal of the lesions, palliative surgery is often associated with the risk of recurrence. Albendazole inhibits glucose uptake and induces glycogen storage depletion of the parasites and finally leads to its death. In this case, *E. multilocularis* invaded the liver and lungs. The patient was conservatively treated according to her wishes. After albendazole anthelmintic treatment, the lung lesions diminished and appeared as cavities. After radiofrequency ablation treatment, the patient’s liver CT revealed that the lesions had decreased persistently. Due to the recovery of the patient’s liver function, curative resection was not performed for further treatment. Thus, systemic anthelmintic drugs are the main therapy for patients with multiple lung metastases.

This report has several limitations. First, liver drainage fluid samples were not collected for mNGS assessment. Second, no other methods, such as polymerase chain reaction or Sanger sequencing, were used to verify the presence of *E. multilocularis*. Finally, a close and long-term follow-up for this patient is required.

In conclusion, this case report illustrates the challenges in diagnosing AE in areas non-endemic to echinococcosis, which is often misdiagnosed as a malignant tumor or metastatic tumor. When the pathogen is unknown and all cultures are negative, mNGS could be a novel approach for fast and accurate diagnosis of a clinically occult infection and could assist clinicians in distinguishing potential pathogens rapidly and efficiently, especially under complicated circumstances.

## Data Availability

The data that support the conclusions of this article are included in this published article. The mNGS sequence results have been deposited in the NCBI database under BioProject accession number SRA: PRJNA1077456.

## Abbreviations

AE: Alveolar echinococcosis
mNGS: Metagenomic next-generation sequencing
CT: Computed tomography
CE: Cystic echinococcosis
